# Supplementary figures and images for: Preservation media, durations and cell concentrations of short-term storage affect key features of human adipose-derived mesenchymal stem cells for therapeutic application
Source: PeerJ. 2017 May 17;5:e3301. doi: 10.7717/peerj.3301 (PMC5437859; doi:10.7717/peerj.3301)

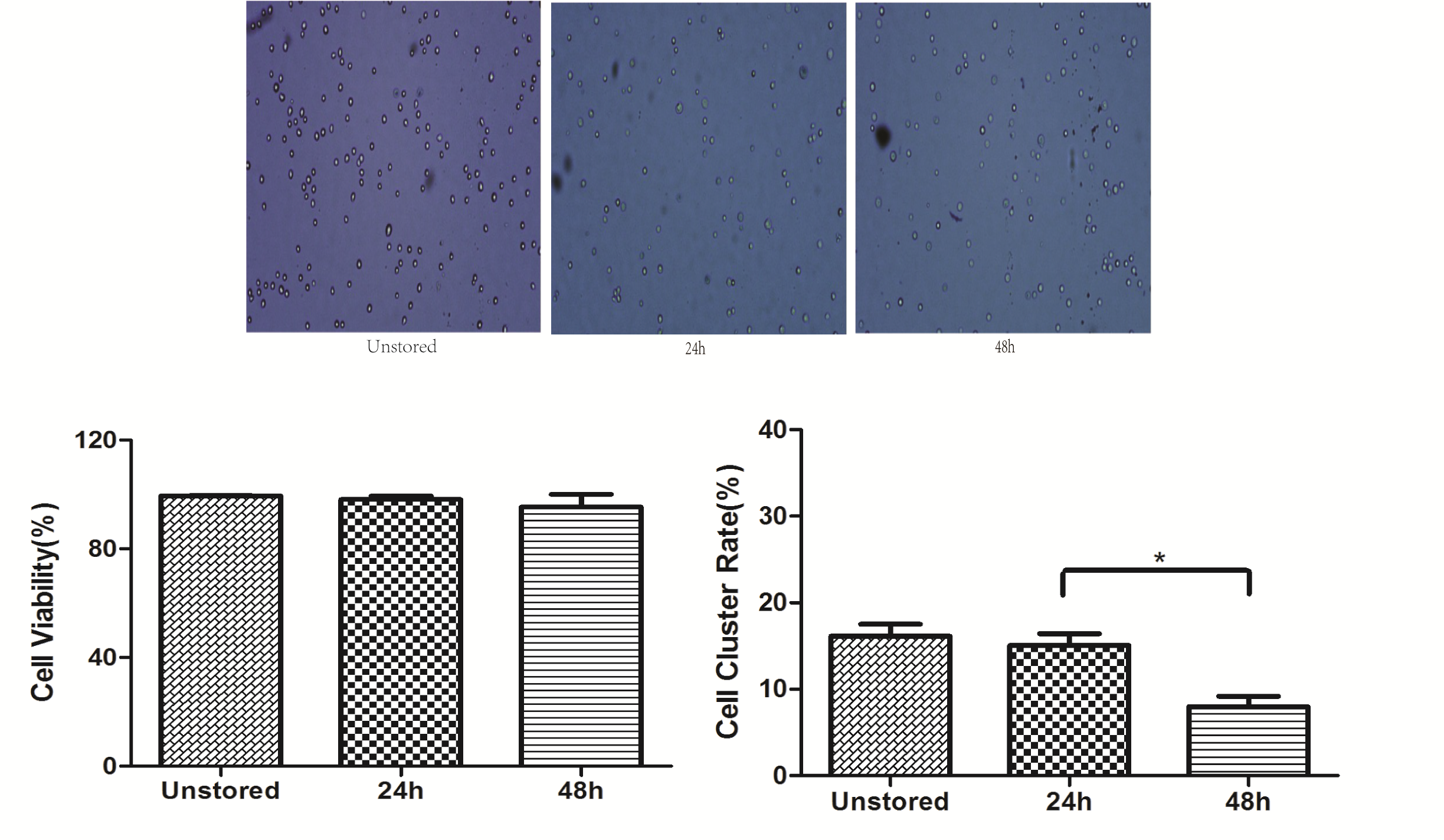

Supplement: Figure S1 — Results were presented as the means ± standard deviation for n = 3, ∗P < 0.05. [file peerj-05-3301-s001.png]
