# Supplementary material for: Preservation media, durations and cell concentrations of short-term storage affect key features of human adipose-derived mesenchymal stem cells for therapeutic application
Source: PeerJ. 2017 May 17;5:e3301. doi: 10.7717/peerj.3301 (PMC5437859; doi:10.7717/peerj.3301)
Supplement: Supplemental Information 1 [file peerj-05-3301-s003.docx]

aatttctcactgcccctgtgataaactgtggtcactggctgtggcagcaactattataagatgctctgaaaactcttcagacactgaggggcaccagaggagcagactacaagaatggcacacgctatggaaaactcctggacaatcagtaaagagtaccatattgatgaagaagtgggctttgctctgccaaatccacaggaaaatctacctgatttttataatgactggatgttcattgctaaacatctgcctgatctcatagagtctggccagcttcgagaaagagttgagaagttaaacatgctcagcattgatcatctcacagaccacaagtcacagcgccttgcacgtctagttctgggatgcatcaccatggcatatgtgtggggcaaaggtcatggagatgtccgtaaggtcttgccaagaaatattgctgttccttactgccaactctccaagaaactggaactgcctcctattttggtttatgcagactgtgtcttggcaaactggaagaaaaaggatcctaataagcccctgacttatgagaacatggacgttttgttctcatttcgtgatggagactgcagtaaaggattcttcctggtctctctattggtggaaatagcagctgcttctgcaatcaaagtaattcctactgtattcaaggcaatgcaaatgcaagaacgggacactttgctaaaggcgctgttggaaatagcttcttgcttggagaaagcccttcaagtgtttcaccaaatccacgatcatgtgaacccaaaagcatttttcagtgttcttcgcatatatttgtctggctggaaaggcaacccccagctatcagacggtctggtgtatgaagggttctgggaagacccaaaggagtttgcagggggcagtgcaggccaaagcagcgtctttcagtgctttgacgtcctgctgggcatccagcagactgctggtggaggacatgctgctcagttcctccaggacatgagaagatatatgccaccagctcacaggaacttcctgtgctcattagagtcaaatccctcagtccgtgagtttgtcctttcaaaaggtgatgctggcctgcgggaagcttatgacgcctgtgtgaaagctctggtctccctgaggagctaccatctgcaaatcgtgactaagtacatcctgattcctgcaagccagcagccaaaggagaataagacctctgaagacccttcaaaactggaagccaaaggaactggaggcactgatttaatgaatttcctgaagactgtaagaagtacaactgagaaatcccttttgaaggaaggttaatgtaacccaacaagagcacattttatcatagcagagacatctgtatgcattcctgtcattacccattgtaacagagccacaaactaatactatgcaatgttttaccaataatgcaatacaaaagacctcaaaatacctgtgcatttcttgtaggaaaacaacaaaaggtaattatgtgtaattatactagaagttttgtaatctgtatcttatcattggaataaaatgacattcaataaataaaaatgcataagatatattctgtcggctgggcgcggtggctcacgcctgtaatcccagcactttgggaggccgaggcgggcggatcacaaggtcaggagatcgagaccatcttggctaacacggtgaaaccccgtctctactaaaaatacaaaaaattagccgggcgcggtggcgggcacctgtagtcccagctactcgggaggctgaggcaggagaatggcgtgaacctgggaggcggagcttgcagtgagccaagattgtgccactgcaatccggcctgggctaaagagcgggactccgtctcaaaaaaaaaaaaaaaaagatatattctgtcataataaataaaaatgcataagatataaaaaaaaaaaaaaa
